# Factors Affecting EWS-FLI1 Activity in Ewing's Sarcoma

**DOI:** 10.1155/2011/352580

**Published:** 2011-11-10

**Authors:** David Herrero-Martin, Argyro Fourtouna, Stephan Niedan, Lucia T. Riedmann, Raphaela Schwentner, Dave N. T. Aryee

**Affiliations:** Children's Cancer Research Institute, St Anna Kinderkrebsforschung, 1090 Vienna, Austria

## Abstract

Ewing's sarcoma family tumors (ESFT) are characterized by specific chromosomal translocations, which give rise to EWS-ETS chimeric proteins. These aberrant transcription factors are the main pathogenic drivers of ESFT. Elucidation of the factors influencing EWS-ETS expression and/or activity will guide the development of novel therapeutic agents against this fatal disease.

## 1. Introduction

ESFT comprise a group of undifferentiated, highly malignant small blue round-cell pediatric tumors. They are genetically characterized by *EWS-ETS* gene rearrangements affecting *EWSR1 *and genes of the ETS-family of transcription factors, predominantly *FLI1* characterizing 85% of cases [1]. Numerous efforts have been undertaken in the last three decades to explore the functional role of EWS-FLI1 in tumor pathogenesis. EWS-FLI1 has been identified as the main genetic factor of malignancy in ESFT [2, 3] and it is also causal in the pathogenesis of ESFT from its cellular types of origin [4–6]. It has been widely documented that this chimeric protein acts both as a transcriptional activator and repressor of similarly sized sets of target genes [7, 8]. While most mechanistic studies have concentrated on the identification and description of downstream EWS-FLI1 regulated genes, this paper focuses on currently known factors influencing EWS-FLI1 activity up- and downstream of the fusion protein and consequently modulate its target gene expression.

## 2. Structure and Posttranslational Modifications Affecting the Transcriptional Activity of EWS-FLI1

Since, due to its tumor-specific expression, EWS-FLI1 protein is considered an ideal therapeutic target [71], significant efforts have been made to understand the function of this fusion protein. Knowledge about the detailed EWS-FLI1 protein structure would be extremely helpful to analyse and predict its DNA-binding properties as a basis for a better understanding of the EWS-FLI1 transcriptional network and for the development of inhibitory modalities with therapeutic promise.

EWS-fusion proteins contain at least the N-terminal 7 exons of *EWS* comprising the EWS activation domain (EAD). The EAD structure consists of multiple degenerate hexapeptide repeats (consensus SYGQQS) with a conserved tyrosine residue. However, systematic mutagenesis of the EAD revealed that the overall sequence composition and not the specific sequence of the degenerate hexapeptide repeat confer EAD activity [72]. The C-terminal portion of EWS-FLI1 consists of a COOH-terminal domain as well as an ets-type winged helix-lop-helix DNA-binding domain (DBD). Arvand et al. suggested that, in addition to the EAD and DBD domains, the COOH-terminal FLI1 domain contributes to promote cellular transformation [73]. Mutation analysis of the EWS-DBD revealed that EWS-FLI1, apparently, not only induces DBD-dependent but also DBD-independent oncogenic pathways, suggesting that EWS-FLI1 interacts with other gene regulatory factors or complexes [74].

Transcriptional regulation is tightly controlled by transcription factor binding to regulatory regions within DNA as well as recruitment of cofactors. Although ETS transcription factors bind predominantly as monomers to a GGAA/T core motif in promoter or enhancer regions of their target genes, functional interaction between ETS proteins and other factors is crucial to enhance or modulate DNA binding [75]. Even though EWS-FLI1 possesses protein interaction domains such as SH2 or PDZ, the identified intrinsically disordered protein regions may facilitate protein-protein complexes as explained in the next chapter [76].

However, transcriptional control also involves complex upstream signaling pathways that converge on the posttranslational modification of transcription factors and their interacting cofactors. Phosphorylation and glycosylation are two examples of posttranslationally modifying mechanisms affecting EWS-FLI1 activity. The EWS portion of about 20% of EWS-FLI1 fusion proteins (those that retain EWS amino acids 256 to 285) contains a conserved calmodulin-binding motif within the IQ domain with a phosphorylated internal Protein Kinase C recognition site at Ser 266 [9]. Mutation of this residue was enough to significantly reduce DNA binding of EWS-FLI1 *in vitro* [9, 10]. Furthermore, EWS and EWS-FLI1 are phosphorylated at Thr 79 in the N-terminal domain in response to DNA damage or mitogens [11]. Glycosylation is the enzymatic process that attaches glycans to proteins, lipids, or other organic molecules [77]. EWS-FLI1 was found to undergo O-linked beta-N-acetylglucosaminylation (O-GlcNAcylation). This modification seems to be reciprocally related to phosphorylation and to influence the transcriptional activation propensities of the fusion protein [12]. In addition, N-linked glycosylation was described as essential to sustain ESFT cell growth. Interestingly, inhibition of N-linked glycosylation decreased the expression of EWS-FLI1 correlating to growth arrest [13]. The highly decreased expression levels of EWS-FLI1 observed after treatment with HMG-CoA reductase inhibitors (i.e., lovastatin) or N-linked glycosylation inhibitors (i.e., tunicamycin) were found to be due to the instability of de novo-synthesized fusion protein [13, 52]. Lovastatin triggered differentiation and induced apoptosis without causing cell cycle arrest through the loss of an RB-regulated G1 checkpoint [52]. Although EWS-FLI1 contains four potential sites for this type of posttranslational modification, no evidence for direct N-glycosylation of the fusion protein could be obtained. Therefore, an indirect functional interaction involving other key-player glycoproteins has been proposed [13]. Since blockage of N-linked glycosylation also leads to inactivation of IGF-1R signaling by inhibiting translocation to the cell surface [14], and since IGF-1R activity is essential to EWS-FLI1 expression (discussed in Section 4), inactivation of this pathway may at least partially explain why inhibition of N-linked glycosylation leads to reduced expression of the fusion protein. However, further investigations are required to test this hypothesis (summarized in Table 1). 

## 3. Direct EWS-FLI1 Protein Interactions

Biochemical purification and analysis identified EWS-FLI1 as an intrinsically disordered protein [72, 78]. Intrinsically disordered proteins are defined by their lack of a stable structure when isolated. A characteristic composition of amino acids prevents these proteins from forming singular, fixed structures thereby enabling them for rapid complex formation and dissociation with relatively high specificity and low affinity [76]. As no direct enzymatic activity has been ascribed to EWS-FLI1, it is necessary to identify interaction partners of the fusion protein in order to learn more about the functional pathways in which it is involved and how to modulate them therapeutically.

EWS-FLI1 is generally perceived as a transcriptional activator [79–81]. Consistent with its transcriptional activator function, EWS-FLI1 associates with several proteins of the basal transcription machinery. Among them are RNA polymerase II [15] and its core subunit hsRBP7 [16–18], CREB-binding protein (CBP)/p300 [19], and RNA helicase A (RHA) [22]. The interaction with (CBP)/p300 was demonstrated to be involved in the regulation of several bona fide EWS-FLI1 targets like p21 [82] or matrix metalloproteinase (MMP-1) [83]. RHA is a modulator of transcription as it interacts with CBP/p300 and RNA polymerase II. Interruption of this interaction induces apoptosis *in vivo* and *in vitro*, a potential novel therapeutic strategy [22, 53]. Interaction with the putative tumor suppressor BARD1, that associates with the breast cancer susceptibility gene BRCA1, links EWS-FLI1 with proteins involved in genome surveillance, DNA repair, and checkpoint control [23]. It is likely that target site selectivity of EWS-FLI1 is mediated via interaction with other sequence specific transcription factors. Such an interaction has been described for FOS-JUN dimers, which bind to AP1 sequences synergizing with EWS-FLI1 in the regulation of a subset of EWS-FLI1 target genes including uridine phosphorylase [24]. Recent *in silico* analyses reveal a significant enrichment of E2F binding sites in EWS-FLI1 upregulated genes suggesting an important role of the E2F family of transcription factors in EWS-FLI mediated transcriptional regulation [8]. Whether EWS-FLI1 actually physically interacts with E2Fs to accomplish upregulation of the affected genes or merely binds alongside E2F transcription factors remains to be elucidated.

In addition to transcriptional activation, an at least equal number of genes are downregulated by EWS-FLI1 as are upregulated [25]. One explanation for this fact is that some of the upregulated EWS-FLI1 targets are transcriptional repressors as exemplified by NKX2.2, a directly EWS-FLI1-activated target which functions as a transcriptional repressor [84, 85]. Another target of EWS-FLI1, NR0B1, not only acts as a transcriptional regulator downstream of EWS-FLI1 but also has recently been shown to interact physically with EWS-FLI1 to influence gene expression thereby contributing to Ewing's sarcoma oncogenesis [25]. Due to interaction with several RNA processing proteins including the small nuclear ribonucleoprotein (snRNP) U1C [86], EWS-FLI1 activity has not only been linked to RNA transcription but also to splicing [26, 87]. U1C plays a critical role in the initiation and regulation of pre-mRNA splicing as part of the U1 small nuclear ribonucleoprotein and commits pre-mRNAs to the splicing process [88]. Interestingly, forced U1C expression was demonstrated to modulate dose—dependently the transcriptional transactivation activity of EWS-FLI1 *in vitro* and *in vivo* via interaction with the EWS amino terminal domain [86]. In addition, experimental evidence for a direct interaction between EWS-FLI1 and EWS was reported by Spahn et al. [20]. Since EWS interacts with a multitude of RNA processing factors [21], the functional consequences of this heterodimerization on RNA splicing remains a subject for further investigation (summarized in Table 1).

## 4. Factors Indirectly Affecting EWS-FLI1 Activity

### 4.1. p53 and INK4A Pathways

The p53 and INK4A (p16/p14ARF) pathways are critical in promoting cell cycle arrest in response to mitogenic signals, and mutations in their key components facilitate tumor progression in most cancer types [89, 90]. In normal primary mouse fibroblasts (MEFs), EWS-FLI1 expression is unstable eliciting a p53-dependent growth arrest and apoptosis program. However, in p16 or p53 defective MEFs, these effects are attenuated and this environment allows stable expression of the fusion protein [27, 28]. Thus, it appears that the loss of each of these tumor suppressor genes stabilizes EWS-FLI1 expression. Consistent with this finding, loss of p53 greatly accelerates tumorigenesis in EWS-FLI1 transgenic mice [29]. However, in ESFT, mutations in *p53 *or *p16/p14ARF *are found in approximately 10% and 25% of cases, respectively. As in most pediatric malignancies, the majority of ESFT express wild-type *p53* and *p16/14ARF* genes [30–32]. Functionally, basal p53 expression is modulated by EWS-FLI1 through an indirect mechanism that involves suppression of the Notch signalling pathway [33].

### 4.2. Hypoxia

Hypoxia is a common condition in solid tumors. It drives cancer cells towards a coordinated set of survival responses altering the transcriptional regulation of many genes [91], stimulating cell migration, invasiveness and motility [92], and driving a metabolic shift towards anaerobic glycolysis [93] or promotion of autophagy [94]. Due to its involvement in drug resistance [95], hypoxia has been identified as a negative prognostic factor in many cancers [96] including sarcomas [97]. HIF-1, a basic HLH transcription factor, is a major player in the adaptive response to hypoxic conditions, enhancing cell survival in this unfavourable environment [92–98]. In ESFT, hypoxia has been shown to contribute to apoptosis resistance via HIF-1*α* [99], to chemotherapy resistance [34], and to the establishment of an alternative circulatory system [35]. Interestingly, under hypoxic conditions, EWS-FLI1 protein expression was demonstrated to increase transiently in a HIF-1*α*-dependent manner [36]. HIF-1*α*-mediated EWS-FLI1 accumulation involved protein regulation at the FLI1 moiety, since the observed protein accumulation was restricted to EWS-FLI1 and neither observed for full-length EWS nor for an alternative EWS fusion to ERG. On the transcriptional level, however, the upregulation of EWS-FLI1 protein did not simply result in a reinforcement of the EWS-FLI1 transcriptional signature, but showed a more complex effect with both synergistic and antagonistic consequences on EWS-FLI1 regulated genes [36]. Another study has shown colocalisation of HIF-1*α* and necrotic areas in an ESFT tissue array, suggesting a role for hypoxia in *in vivo* induction of HIF-1*α* [37]. Data thus implicates HIF as the main response factor for hypoxic stimulus in ESFT with marked effects on proliferation and apoptosis. 

### 4.3. IGF-1/IGF-1R and bFGF Pathways

The autocrine loops encompassing (IGF-1)/(IGF-1R) and (IGF-2)/(IGF-2R) play a crucial role in the proliferation and survival of ESFT cells via activation of AKT and ERK1/2 [38–40]. Notably, in MEFs, expression of IGF-1R is required for EWS-FLI1-mediated cellular transformation suggesting that the oncogenic activity of the fusion protein is dependent on functional IGF-1R signaling [41]. There are several lines of evidence that support a link between EWS-FLI1 and IGF-1/IGF1-R signalling [42, 43] also in one of the putative progenitor cell of ESFT [44], and inhibition of this signaling pathway reduces tumor growth *in vitro* [45] and *in vivo* [54], blocks angiogenesis [55], induces cell death [61], and increases chemosensitivity [100]. 

A further growth factor positively interacting with EWS-FLI1 activity is basic fibroblast growth factor (bFGF). bFGF was demonstrated to trigger EWS-FLI1 expression in serum-depleted ESFT cells. A neutralizing antibody against bFGF was able to disrupt this upregulation and inhibit expression of the fusion protein in a broad panel of ESFT cell lines [46]. No detectable effect on EWS-FLI1 expression levels was observed upon epidermal growth factor or platelet derived growth factor stimulation. However, the mechanism by which bFGF specifically controls EWS-FLI1 levels remains elusive 

Most recently, a further putative signalling molecule that is expressed on the cell surface, the bladder cancer associated protein BLCAP, carrying a putative Ser-Pro-X-X motif and a proline-rich area, was reported to modulate EWS-FLI1 expression [47]. The mechanism of this activity, which was obtained upon artificial ectopic overexpression, remains to be elucidated (summarized in Table 1).

## 5. miRNAs Influencing EWS-FLI1 Activity

MicroRNAs (miRNAs) are small (21–24 nucleotides), single-stranded, and noncoding RNAs that regulate gene expression in a variety of cellular processes [101]. By binding of the miRNA to a partially homologous region (seed region) within the 3′ untranslated region (UTR), coding sequences or 5′UTRs of messenger RNAs (mRNA), it can either block its target mRNA translation or lead to its degradation [102, 103]. Due to the imperfect base pairing of the miRNA to its seed region, a single miRNA can regulate several target mRNAs as part of a complex gene regulatory network [101, 104]. It is estimated that between 30% and 60% of the human genome is regulated by miRNAs including genes involved in mechanisms of tumorigenesis, such as proliferation, inflammation, stress response, apoptosis, differentiation, and invasion [101, 102]. miRNAs can either act as oncogenes or tumor suppressors, some of them even in both ways [101, 105, 106]. 

While the role of aberrantly expressed miRNAs is well established in adult cancers, only few studies exist for pediatric malignancies in general and sarcomas in particular [107–109]. One of the best described tumor suppressive miRNAs is miR-145, which was found to be downregulated in several solid tumors, including lung, colorectal, breast, and prostate cancer [110, 111]. Similarly, in ESFT, miR-145 was recently described as the top consistently EWS-FLI1 repressed miRNA. This finding was based on the investigation of five ESFT cell lines upon RNA interference-mediated EWS-FLI1 knockdown and on differential gene expression patterns between primary ESFT and mesenchymal stem cells, the most related normal tissue. In fact, miR-145 and EWS-FLI1 were demonstrated to build a regulatory feedback loop, in which EWS-FLI1 suppresses miR-145 and miR-145 modulates EWS-FLI1 expression [48, 49]. This type of positive feedback regulation has the potential to serve as a compensating buffer for variations in EWS-FLI1 expression. Reconstitution of miR-145 expression resulted in decreased EWS-FLI1 expression and consequently reduced cell growth and soft agar colony formation [48]. Of note, miR-145 has recently been reported to target the 3′UTR of another ETS family gene, ERG, which replaces FLI in alternative EWS fusions associated with about 10% of ESFT [112]. The DNA binding domain of ERG shares 98% homology with that of FLI1 [113] and our own unpublished results suggest that there is significant overlap between EWS-FLI1 target genes in ESFT and ERG in prostate cancer cells. Although activity of miR-145 on EWS-ERG in ESFT remains to be demonstrated, the finding of ERG modulation by this miRNA in prostate cancer cells may extend the concept of feedback regulation between EWS-ETS fusion genes and miR-145 beyond EWS-FLI1. 

However, a recent global miRNA profiling study in the A673 ESFT cell line did not confirm miR-145 among EWS-FLI1 suppressed miRNAs but described a group of EWS-FLI1 repressed miRNAs (miR-100, miR-125b, miR-22, miR-221/222, miR-271, and miR29a) with predicted targets in the IGF-1/IGF-1R signaling pathway [50], a key growth regulatory signaling pathway interacting with EWS-FLI1 expression/activity [41–43]. The lack of evidence for miR-145 suppression in this study [50] as compared to the previous study [48] may be caused by the use of different cell lines, different screening platforms (Agilent-type micro-array versus Applied Biosystems quantitative stem loop PCR), and/or the different timing of miRNA screening after EWS-FLI1 knockdown (10 days in [50] versus 4 days in [48]). miR-145 is the first miRNA shown to target FLI1 and FLI1 fusion genes [48, 49, 110]. Given the length of the FLI1 3′UTR (>2 kb), it is very likely that other miRNAs may have similar FLI1 and EWS-FLI1 modulatory activities (summarized in Table 1).

## 6. Therapeutic Potentials

The existence of tumor-specific alterations in several cancers presents a unique opportunity for pharmacological intervention to therapeutic benefit. Although EWS-FLI1 has only been identified in tumor cells and therefore provides a potential ideal therapeutic target, ESFT has so far remained a targetable disease without a targeted drug [71, 114]. Suppression of EWS-FLI1 has been achieved by antisense technologies [115–121], small interfering RNA (siRNA) [122–125], short hairpin RNA (shRNA) [42, 126–128], and small pharmacological compounds [53, 62] all blocking the proliferation of ESFT cell lines and xenografted tumors. Although some siRNA coupled to nanoparticles have proved to be useful in preclinical models either alone [129–132] or combined with other therapeutic agents as rapamycin [133], the general lack of clinical translation of some of these macromolecule-based strategies lies in the challenge of pharmacological delivery [134]. Being present only in tumor cells, directly targeting the activity of EWS-FLI1 by focusing on its protein-protein interactions, will be a logical step towards identifying potential targets for developing effective anti-ESFT therapies. Along this line, targeting binding partners essential for EWS-FLI1 oncogenic function holds promise in combating ESFT as has been shown for RNA helicase A using the small molecule YK-4-279 [53]. YK-4-279 blocked RNA helicase A binding to EWS-FLI1, induced apoptosis in ESFT cell lines and also reduced growth in ESFT xenografts. YK-4-279 can also target a subpopulation of chemoresistant ESFT stem cells [135] and it has been recently described as an effective antiinvasive agent in ETV1 and ERG fusion positive prostate tumors although the mechanism of action of YK-4-279 in prostate cancer cells seems to be different [136]. A further evaluation of this new role of YK-4-279 in ESFT would be needed. O-linked beta-N-acetylglucosamine (O-GlcNAc), which modifies nuclear and cytoplasmic proteins on serine and threonine residues, was delineated to serine/threonine residues of the amino-terminal EWS transcriptional-activation domain of the EWS-FLI1 fusion protein by our laboratory. Inhibition of EWS-FLI1 O-GlcNAcylation interfered with transactivation of its target gene *Id2 *[12]. A better understanding of EWS-FLI1 O-GlcNAcylation as it relates to gene transcription and the physiological mechanisms behind this process is likely to lead to novel therapies for treating ESFT. Recently, our group identified a positive feedback regulation between EWS-FLI1 and miR-145 as an important component of EWS-FLI1 mediated tumorigenesis [48]. As such, targeting miR-145 or other miRNAs found to affect EWS-FLI1 activity may serve as a promising therapy strategy to improve the clinical outcome of ESFT patients. Also, the adaptation of tumors to hypoxia is critical for their survival and growth. Given the central role hypoxia plays in tumor progression and resistance to therapy, hypoxia might well be considered the best validated target that has yet to be exploited in oncology [137]. Some established drugs targeting hypoxia or the HIF-1 pathway (e.g., 2-methoxyestradiol, bortezomib) have been already tested in ESFT [63–65] and although bortezomib per se showed no clinical benefit [138] and resistance appeared, [139] the recent finding of hypoxia transiently enhancing EWS-FLI1 protein expression [36] may raise hopes for a combined therapeutic window for ESFT patients with new agents. Also, therapeutic strategies targeting the IGF-1/IGF-1R loop may interfere with oncogenic functions of EWS-FLI1. Antagonistic IGF-1R antibodies or small kinase inhibitory molecules have been developed and are therefore currently tested in phase I/II clinical trials on ESFT patients either alone [56–58] or in combination with the mTOR inhibitor temsirolimus [59] showing promising results. One important fact is the status of the insulin receptor (IR) as ESFT patients with a low IGF-1R : IR ratio do not benefit from anti-IGF-1R therapies [60]. A meta-analysis of small-scale retrospective studies suggest that, although rare, ESFT harbouring *p53 *or *p16/p14ARF *mutations form a subset with particularly poor prognosis, highly aggressive behaviour, and poor chemoresponse [140, 141]. Nutlin-3a, a small molecule which antagonizes the interaction of MDM2 with p53, thus stabilizing the tumor suppressor protein, is able to promote a strong apoptotic arrest when applied to ESFT and showed a synergistic effect with other chemotherapeutic agents such as etoposide, doxorubicin, vincristine, and actinomycin D in a dose-dependent manner [66, 67]. As downstream targets of EWS-FLI1 have been reported to contribute to the oncogenic activities of EWS-FLI1 [19, 25, 85, 126, 142–145], generating compounds effectively targeting these downstream effectors hold potential therapeutic benefits as has been shown with ET-743 [68], Mithramycin [62], and ARA-C [69] although it is necessary to be cautious as, for example, ARA-C has shown minimal activity and hematologic toxicity in a phase II clinical trial [70]. Methods to evaluate the specificity, toxicity, metabolism, and excretion as well as adsorption and distribution within tumor cells are warranted to advance these potential drugs into clinical trials (summarized in Table 2).

## 7. Conclusion

While attempts to understand the pathobiology of ESFT have focused mainly on identifying EWS-FLI1 target genes and downstream pathways, there are still many important unresolved questions regarding factors modulating EWS-FLI1 activity. Manipulation of these factors may offer therapeutic promise since it is difficult to directly target a transcription factor. This may be achieved by applying high throughput compound screening technologies as has been performed to block EWS-FLI1 interaction with RNA helicase A [22, 53], and in EWS-FLI1 signature-based approaches as in the case of Mithramycin [62]. Such compounds may be more specific and highly effective in neutralising EWS-FLI1 activity in ESFT cells with minimal toxicity.

The list of agents influencing EWS-FLI1 fusion protein activity and/or expression is consistently enriched. Some of them show crosstalk, as has been demonstrated between a group of EWS-FLI1 repressed miRNAs and targets of IGF-1/IGF-1R pathway [50]. Since very little is known about the influence of miRNAs on EWS-FLI1 activity, employing high-throughput screening assays to identify miRNAs with specific effects on EWS-FLI1 activity will provide additional targets for therapeutic development. Recently, the use of miRNA arrays to compare the miRNA expression profile of human mesenchymal stem cells (MSCs) and ESFT cell lines has shown induction of the oncogenic miRNA 17–92 cluster and repression of the tumor suppressor let-7 family. Importantly, the feasibility of delivery of synthetic miRNA *in vivo* to achieve tumor growth inhibition was demonstrated in this study [51]. For a better understanding of the interplay between the discussed factors, it should be crucial to also consider the individual clinical profiles of ESFT patients.

## Figures and Tables

**Table 1:** Factors influencing EWS-FLI1 activity and/or expression.

*Posttranslational modifications*		
Phosphorylation	DNA binding, response to DNA damage and mitogens	[9–11]
Glycosylation	Transcriptional activation, cell growth, link with IGF-1 signaling	[12–14]

*Direct protein-protein interactions*		
RNA polymerase II	Basal transcription machinery	[15]
hsRBP7	Basal transcription machinery	[16–18]
Creb-binding protein (CBP)/p300	Basal transcription machinery, regulation of EWS-FLI1 targets like p21 or MMP-1	[19–21]
RNA helicase A (RHA)	Modulator of transcription	[22]
BARD1	Putative tumor suppressor; genome surveillance, DNA repair and checkpoint control	[23]
FOS-JUN dimers	Binding to AP1 sequences synergizing with EWS-FLI1, regulation of uridine phosphorylase	[24]
NR0B1	Transcriptional regulator downstream of EWS-FLI1	[25]
small nuclear ribonucleoprotein (snRNP) U1C	pre-mRNA splicing	[26]
EWS	Functional consequences of this heterodimerization unknown	[20]

*Factors indirectly affecting EWS-FLI1 activity*		
p53 and INK4A pathways	Loss of each one stabilizes EWS-FLI1	[27–33]
Hypoxia	Apoptosis resistance via HIF, chemotherapy failure, angiogenesis	[34–37]
IGF-1/IGF-1R pathway	EWS-FLI1 mediated cellular transformation, proliferation and survival	[38–45]
bFGF	Triggers EWS-FLI1 expression in serum-depleted ESFT cells	[46]
BLCAP	Ectopic overexpression decreases EWS-FLI1, apoptosis	[47]

*miRNAs*		
miR-145	EWS-FLI1 repressed miRNA, regulatory feedback loop	[48, 49]
miR-100, miR-125b, miR-22, miR-221/222, miR-271 and miR29a	EWS-FLI1 repressed miRNAs, targets in IGF signalling pathway	[50]
let-7 family	EWS-FLI1 repressed miRNA, let 7-a is a direct target of EWS-FLI1	[51]
miRNA 17–92 cluster	EWS-FLI1 induced miRNAs	[51]

**Table 2:** Therapeutic agents targeting partners essential for EWS-FLI1.

Name	Characteristics	Effects	Reference
Mevalonate, tunicamycin	Inhibitors of N-linked glycosylation	EWS-FLI1 expression, growth arrest, inactivation of IGF-1R signaling	[12–14]
Lovastatin	HMG-CoA reductase inhibitor	Triggering of differentiation, induction of apoptosis, inactivation of IGF-1R signaling	[14, 52]
YK-4-279	Blocking RNA helicase A binding to EWS-FLI1	Induction of apoptosis *in vitro* and reduction of growth *in vivo *	[53]
Anti-IGF-1R antibodies	Blocking IGF-1/IGF-1R pathway	Tumor growth reduction *in vitro* and *in vivo*, angiogenesis blockage, cell death induction and chemosensitivity increase	[45, 54–60]
Epigallocatechin gallate	IGF-1R inhibitor, catechin derivative	Blocks proliferation and induces cell death	[61]
Neutralizing antibody against bFGF	Blocking bFGF pathway	EWS-FLI1 downregulation through inhibition of FGFR phosphorylation	[46]
Mithramycin	DNA binding transcriptional inhibitor	EWS-FLI1 inhibitor, decreases tumor growth *in vitro* and *in vivo *	[62]
2-methoxyestradiol, bortezomib	Inhibitors of hypoxia and/or HIF-1 pathway	Induction of apoptosis, autophagy and cell cycle arrest *in vitro *	[63–65]
Nutlin-3a	Small molecule which antagonizes the interaction of MDM2 with p53	Stabilization of p53, apoptotic arrest, synergistic effect with other chemotherapeutic agents	[66, 67]
Ecteinascidin 743	Binds and alkylates DNA at the N2 position of guanine	Induction of apoptosis, reduction of the activity of EWS-FLI1 targets	[68]
ARA-C (cytosine arabinoside)	Antimetabolite, inhibitor of EWS-FLI1	EWS-FLI1 protein reduction, decrease of cell viability, transformation and tumor growth *in vivo *	[69, 70]
Synthetic Let-7a	Synthetic miRNA	Restored Let-7a expression resulted in ESFT growth inhibition *in vivo *	[51]

## Abbreviations

ESFT: Ewing's sarcoma family tumors
EAD: EWS activation domain
DBD: DNA-binding domain
SH2: Src Homology 2
O-GlcNAcylation: O-linked beta-N-acetylglucosaminylation
HMG-CoA: 3-Hydroxy-3-methylglutaryl-coenzyme A
RB: Retinoblastoma
IGF-1: Insulin-like growth factor 1
IGF-1R: Insulin-like growth factor receptor 1
CBP: Creb-binding protein
RHA: RNA helicase A
MMP-1: Matrix metalloproteinase
BARD1: BRCA1-associated RING domain protein 1
snRNP: Small nuclear ribonucleoprotein
MEFs: Mouse embryonic fibroblasts
HIF-1: Hypoxia-inducible factor-1
bFGF: Basic fibroblast growth factor
IGF-2: Insulin-like growth factor 2
IGF-2R: Insulin-like growth factor receptor 2
miRNA: MicroRNA
UTR: 3′ untranslated region
mRNA: Messenger RNA
shRNA: Short hairpin RNA
O-GlcNAc: O-linked beta-N-acetylglucosamine
ARA-C: Cytosine arabinoside
IR: Insulin receptor
MSCs: Human mesenchymal stem cells
BLCAP: Bladder cancer-associated protein.
